# The Correlation between Serum Level of Vitamin D and Outcome of Sepsis Patients; a Cross-Sectional Study

**Published:** 2019-01-10

**Authors:** Majid Shojaei, Anita Sabzeghabaei, Helia Valaei Barhagh, Soheil Soltani

**Affiliations:** 1 Emergency Department, Imam Hossein Hospital, Shahid Beheshti University of Medical Sciences, Tehran, Iran.; 2 Emergency Department, Shohadaye Tajrish Hospital, Shahid Beheshti University of Medical Sciences, Tehran, Iran.

**Keywords:** Systemic inflammatory response syndrome, sepsis; patient outcome assessment, 24, 25-dihydroxyvitamin d 3, mortality

## Abstract

**Introduction:**

The effect of vitamin D deficiency in manifestation of sepsis and its role as an important mediator in the immune system has received attention. The present study was done with the aim of evaluating the correlation between serum levels of vitamin D and outcome of sepsis patients.

**Methods:**

The present cross-sectional study was performed on patients over 18 years of age suspected to sepsis presenting to an emergency department during 1 year using non-probability convenience sampling. For all eligible patients, blood sample was drawn for measurement of serum level of vitamin D3 and the correlation of this vitamin with outcomes such as mortality, renal failure, liver failure and etc. was assessed.

**Results:**

168 patients with the mean age of 70.8 ± 13.3 (43.0 – 93.0) years were studied (56.0% male). Mean serum level of vitamin D3 in the studied patients was 19.03 ± 13.08 (4.0 – 85.0) ng/ml. By considering 20 – 50 ng/ml as the normal range of vitamin D, 61.6% of the patients had vitamin D deficiency. Only age (r=-0.261, p=0.037) and mortality (r=-0.426, p=0.025) showed a significant correlation with mean vitamin D. Sepsis patients with older age and those who died had a lower level of vitamin D. Area under the ROC curve of serum vitamin D level regarding 1-month mortality of the sepsis patients was 0.701 (95%CI: 0.439 – 0.964).

**Conclusion:**

Based on the results of the present study, the prevalence of vitamin D deficiency in sepsis patients presenting to the ED was estimated as 61.6%. A significant and indirect correlation was found between the serum level of vitamin D3 and mortality as well as older age. It seems that consumption of vitamin D supplements might be helpful in decreasing the prevalence of infection, sepsis, and mortality caused by it, especially in older age.

## Introduction

Sepsis is a clinical syndrome caused by immune response to various infections. Despite the existence of proper antimicrobial and palliative care, due to the high prevalence of multiple organ failure, being affected with sepsis is associated with a high rate of mortality. More than 31 million sepsis cases, 19 million severe sepsis cases, and 5.3 million deaths a year is a rough estimation of the burden of this disease in high-income countries (1-3).

Recent studies have shown the correlation between vitamin D deficiency and severe infections and sepsis. In addition, it has been confirmed that vitamin D is an important mediator in the immune system and its inhibitory role in sepsis pathogenesis has been evaluated (4-6). Vitamin D can regulate acquired and innate immune responses (7).

This vitamin prevents overexpression of inflammatory cytokines and is an important mediator in aggregation of leukocytes, formation of local inflammation, and anti-bacterial responses in innate immunity. Vitamin D deficiency has been linked to an increase in the risk of initiation and development of viral and bacterial infections (8-10). Low serum level of vitamin D on admission of patients to the intensive care unit has correlated with an increase in mortality risk and blood infections (11). Additionally, a higher risk of developing sepsis has been shown in patients who have had vitamin D deficiency before hospital admission (7, 10).

However, studies in this regard are still ongoing to reach a final decision and the correlation between vitamin D and decrease in mortality has not been confirmed in all studies (12-14). Iran is among the countries that have a considerable prevalence of vitamin D deficiency in all age groups. Iran’s ministry of health has strongly advised using vitamin D supplements for all age groups (15, 16). On the other hand, we are faced with a day by day increase in the number of sepsis patients in emergency departments (ED) and intensive care units. Therefore, the present study was designed and performed with the aim of evaluating the correlation between serum levels of vitamin D and outcome of sepsis patients presenting to ED.

## Methods

Study design and setting

The present cross-sectional study was performed on patients suspected to sepsis presenting to the ED of Imam Hossein Hospital, Tehran, Iran, from 2015 to 2016. For all the eligible patients, blood sample was drawn for measuring serum level of vitamin D and finally the correlation between the level of this vitamin and the studied outcomes was evaluated. The study was performed by adhering to the principles of Helsinki Declaration and approval of the ethics committee of Shahid Beheshti University of Medical Sciences. Inclusion of patients was done after obtaining informed consent from the patient or their relative. Keeping patient data confidential and publishing findings anonymously were among other measures for ethical conduct in this study.

Participants

The studied sample included patients over 18 years of age suspected with sepsis presenting to the emergency department in various working shifts, who were selected via non-probability convenience sampling. Sepsis diagnosis was confirmed after clinical examinations, meeting the required criteria and performance of laboratory tests. Patients with a history of neuroendocrine diseases, history of mental disorders, shock due to reasons other than sepsis, consumption of corticosteroid drugs, recent and constant use of vitamin D or other supplements including it, pregnant women, those who did not give their consent for participation in the study, and those whose sepsis diagnosis was not confirmed in the end based on the mentioned criteria were eliminated from the study.

Sepsis was defined as the presence of at least 2 of the symptoms of systemic inflammatory response syndrome (SIRS) with the origin of a suspected or confirmed infectious source (17).

Sampling

Venous blood was drawn from the median cubital vein of the patients for measuring the serum level of vitamin D. Blood samples were kept in room temperature for 15-30 minutes and centrifuged (1500 rpm) for 10-15 minutes. Consequently, serums were separated and kept in -20°C until the time of evaluation. After completion of samples, the concentration of serum 25-hydroxy vitamin D3 was measured. Measurement of serum concentration of 25-hydroxy vitamin D3 was done with radioimmunoassay method using DIASource kit made in Belgium and based on the guidelines of the kit.

Data gathering 

Data gathering was done by a trained senior emergency medicine resident using a pre-designed checklist including demographic data (age, sex), laboratory findings, vital signs, level of consciousness, origin of the infection, sepsis severity based on Mortality in Emergency Department Sepsis (MEDS) score, and finally, the outcome of patients (death, renal failure, liver failure, need for mechanical ventilation and etc.). 30-day outcome of the patients (mortality) was gathered by follow-up via phone calls.

Based on MEDS score, the patients were divided into 5 groups based on the probability of death including: 1- very low probability, 2- low probability, 3- moderate probability, 4- high probability, and 5- very high probability.

Statistical analysis

Sample size required for the present study was determined to be 195 cases, based on the study by Rech et al. (6), and considering 54% prevalence of vitamin D deficiency in sepsis patients, 95% confidence interval (CI) and maximum acceptable error in estimation of prevalence as 7%. To describe data, frequency and percentage or mean ± standard deviation were reported. To assess the correlation between various factors and vitamin D deficiency, chi-square, Fisher’s exact and ANOVA tests were applied. All analyses were performed using SPSS 21.0 statistical software. Calculation of area under the receiver operating characteristic (ROC) curve was applied for determining the cut-off point of serum level of vitamin D in correlation with mortality. In this study, p<0.05 was considered as the level of significance.

## Results

**Figure 1:**
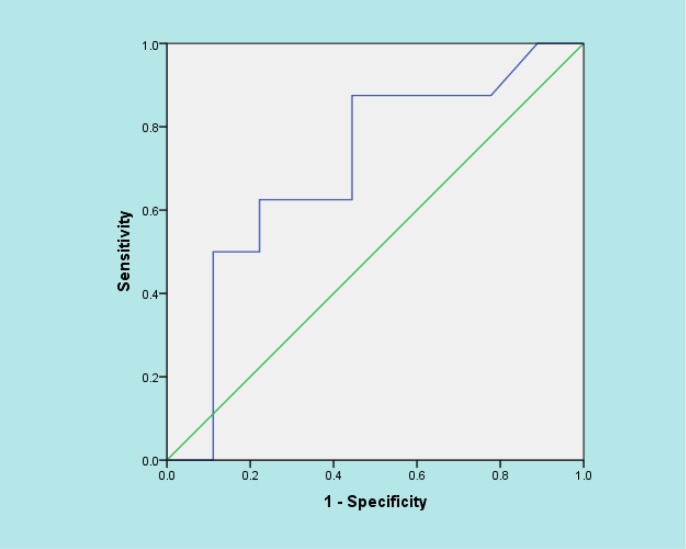
Area under the curve of serum level of vitamin D and 1-month mortality of the sepsis patients presenting to emergency department

168 patients with the mean age of 70.8 ± 13.3 (43.0 – 93.0) years were studied (56.0% male). Tables 1 and 2 depict the baseline characteristics and laboratory findings of the studied patients. Mean serum level of vitamin D3 in the studied patients was 19.03 ± 13.08 (4.0 – 85.0) ng/ml. By considering 20 – 50 ng/ml as the normal range of vitamin D, 61.6% of the patients had vitamin D deficiency. CRP (p=0.176) and platelet (p=0.951) levels, as 2 important inflammatory indices, were not significantly different between the patients with vitamin D deficiency and others. Table 3 shows the correlation between mean serum level of vitamin D and different variables. Only age (r=-0.261, p=0.037) and mortality (r=-0.426, p=0.025) showed a significant correlation with mean serum vitamin D level. Sepsis patients with older age and those who died had a lower level of vitamin D. Area under the ROC curve of vitamin D regarding 1-month mortality of the sepsis patients was 0.701 (95%CI: 0.439 – 0.964) (figure 1). Patients whose vitamin D level was less than 25 ng/ml were significantly more at risk of death (p=0.016). Relative risk of mortality in patients with a vitamin D level less than 25 ng/ml was estimated to be 1.42 (95%CI: 1.027- 1.970).

## Discussion

Based on the results of the present study, the prevalence of vitamin D deficiency among sepsis patients presenting to the ED was estimated as 61.6%. A significant indirect correlation was observed between the serum level of vitamin D and mortality as well as older age.

**Table 1:** Baseline characteristics of studied patients

**Variable**	**Value**
**Sex**	
Male	94.0 (56.0)
Female	74.0 (44.0)
**Age (year)**	
40 – 60	39 (23.0)
≥ 60	129 (77.0)
**Vital signs**	
Systolic blood pressure (mmHg)	89.41 ± 18.63
Pulse rate (/minute)	103.6 ± 9.5
Temperature (C)	38.4 ± 0.7
Saturation O2 (%)	85.1 ± 9.4
**GCS**	
3 - 7	16 (9.6)
8 - 14	87 (50.7)
15	67 (39.7)
**Source of infection**	
Pneumonia	46 (31.1)
Urinary tract infection (UI)	44 (29.7)
Pneumonia + UI	32 (21.6)
Bed sore	14 (9.5)
Multi source	10 (6.9)
Gastrointestinal	2 (1.2)
**Mechanical ventilation**	
Yes	56 (43.1)
No	74 (56.9)
**Septic shock**	
Yes	28 (26.9)
No	76 (73.1)
**30-day mortality**	
Yes	106 (63.1)
No	62 (36.9)
**MEDS score**	
1	31 (18.5)
2	25 (14.9)
3	62 (36.9)
4	33 (19.6)
5	17 (10.1)

Data are presented as mean ± standard deviation or number (%). There is missing data in some variables. GCS: Glasgow coma scale.

**Table 2:** Laboratory findings of studied patients

**Variable**	**Mean ± SD**
pH	7.38 ± 0.08
HCO3 (mEq/L)	23.81 ± 6.58
PaCO2 (mmHg)	38.52 ± 11.51
Hemoglobin (g/dl)	11.02 ± 2.21
Hematocrit (%)	34.21 ± 7.90
White blood cell (10^9^/L)	12256.46 ± 8597.33
Platelet count (10^9^/L)	234675.67 ± 136736.51
Polymorphonuclear (%)	80.62 ± 8.33
Blood urea nitrogen (mg/dL)	81.59 ± 47.09
Creatinine (mg/dL)	1.49 ± 1.26
Aspartate aminotransferase (U/L)	44.61 ± 89.99
Alanine aminotransferase (U/L)	53.77 ± 109.80
Alkaline phosphatase (U/L)	266.41 ± 134.47
Bilirubin total (mg/dL)	0.77 ± 0.40
Bilirubin direct (mg/dL)	0.34 ± 0.28
Vitamin D3 (ng/ml)	19.03 ± 13.08
C-reactive protein (mg/dL)	66.69 ± 28.57

SD: standard deviation.

**Table 3:** Relationship between mean serum vitamin D3 level and different factors

**Variable**	**Vitamin D3**	**P**
**Sex **		
Male	18.67 ± 13.62	0.726
Female	19.43 ± 12.54	
**Age (year)**		
40 – 60	14.44 ± 7.08	0.037
≥ 60	20.18 ± 14.40	
**30-day mortality**		
Yes	17.4 ± 9.9	0.025
No	21.4 ± 11.5	
**Source of infection**		
Pneumonia	20.3 ± 13.0	0.825
Urinary tract infection (UI)	18.1 ± 17.0	
Pneumonia + UI	18.1 ± 9.4	
Bed sore	18.1 ± 10.1	
others	18.3 ± 3.4	
**MEDS score**		
1	18.9 ± 7.7	0.642
2	21.6 ± 11.2	
3	18.4 ± 11.7	
4	18.8 ± 11.5	
5	16.7 ± 8.8	
**Septic shock**		
Yes	21.53± 4.70	0.375
No	18.83 ± 12.87	
**Mechanical ventilation**		
Yes	20.09 ± 15.73	0.428
No	18.01 ± 12.71	
**Acute renal failure (Cr>1.6 mg/dl)**		
Yes	21.92 ± 18.20	0.109
No	17.95 ± 10.66	
**Liver failure (AST>45 U/L)**		
Yes	20.07 ± 15.87	0.613
No	18.66 ± 12.86	

**MEDS:** Mortality in Emergency Department Sepsis.

In line with the present study, previous studies have suggested that vitamin D concentration correlates with the rate of mortality in patients (4-6). In the study by Parekh et al. there was a significant correlation between mean serum level of vitamin D in patients with sepsis who died and those who survived (18).

The results of a study by Rech et al. showed that vitamin D deficiency in patients with severe sepsis or septic shock was significantly associated with increase in 30-day mortality (6). In the study by Nguyen et al. also low serum level of vitamin D was considered related to increase in 23-day mortality among 91 patients with sepsis (5). 

In Arnson et al. study the correlation between vitamin D deficiency and mortality rate was confirmed in critically ill patients (11). In 2399 patients, vitamin D deficiency from 365 days before hospitalization was reported as a reliable predictor for both short term and long term mortality and positive blood culture for microorganism growth (19).

The correlation between sufficient vitamin D and reduced mortality has not been reported in all studies (13, 14). In one study, the rate of mortality in sepsis patients correlated with vitamin D level upon admission, but this correlation was not significant (20).

Based on the National Health and Nutrition Examination Survey, level of 25-hydroxy vitamin D had an inverse correlation with occurrence of upper respiratory tract infection (21). The evidence is also indicative of the effect of vitamin D level on influenza and invasive pneumococcal disease (22). Two meta-analyses performed on observational studies have confirmed that there is a significant correlation between vitamin D status and risk of being affected with sepsis, infection severity and 30-day mortality (23, 24).

An important limitation present in all observational studies is also present in our study and it is not known if vitamin D deficiency is a marker for sepsis or if it causes sepsis, in other words if vitamin D deficiency is the cause for sepsis or its effect.

In an experimental study on mice the causality of vitamin D as the reason for mortality in sepsis was evaluated. The findings of this experimental study showed that imposing vitamin D deficiency on the mice through their diet led to sepsis and increase in inflammation and cellular disorders and bacterial growth as well as disturbing the balance of apoptotic neutrophil aggregation. These experimental studies support the hypothesis that vitamin D deficiency might be a major cause in manifestation of sepsis (18, 25). It seems that with all the present evidence, consumption of vitamin D supplement might be able to reduce infection, sepsis and mortality caused by it, especially in older ages.

Limitations

Controlling all the probable confounding factors in observational studies such as the present one is not possible and this can affect the findings of the study. In addition, this study was a single centered one with a relatively small sample size, which makes data generalization hard. Another limitation of the study was that some data affecting the level of vitamin D such as cytokine level could not be recorded.

## Conclusion:

Based on the results of the present study, the prevalence of vitamin D deficiency in sepsis patients presenting to the ED was estimated as 61.6%. A significant and indirect correlation was found between the serum level of vitamin D and mortality as well as older age. It seems that consumption of vitamin D supplements might be helpful in decreasing the prevalence of infection, sepsis, and mortality caused by it, especially in older age.
